# Evaluation of protective efficacy induced by different heterologous prime-boost strategies encoding triosephosphate isomerase against *Schistosoma japonicum* in mice

**DOI:** 10.1186/s13071-017-2036-5

**Published:** 2017-02-28

**Authors:** Yang Dai, Song Zhao, Jianxia Tang, Yuntian Xing, Guoli Qu, Jianrong Dai, Xiaolin Jin, Xiaoting Wang

**Affiliations:** 1 Key Laboratory of National Health and Family Planning Commission on Parasitic Disease Control and Prevention, Jiangsu Provincial Key Laboratory on Parasite and Vector Control Technology, Jiangsu Institute of Parasitic Diseases, Wuxi, Jiangsu Province 214064 People’s Republic of China; 2 Public Health Research Center, Jiangnan University, Wuxi, Jiangsu Province 214122 People’s Republic of China

**Keywords:** *Schistosoma japonicum*, Vaccination, Heterologous prime-boost strategy, Triosphosphate isomerase, Protective efficacy

## Abstract

**Background:**

In China, schistosomiasis japonica is a predominant zoonotic disease, and animal reservoir hosts in the environment largely sustain infections. The development of transmission-blocking veterinary vaccines is urgently needed for the prevention and efficient control of schistosomiasis. Heterologous prime-boost strategy is more effective than traditional vaccination and homologous prime-boost strategies against multiple pathogens infection. In the present study, to further improve protective efficacy, we immunized mice with three types of heterologous prime-boost combinations based on our previously constructed vaccines that encode triosphate isomerase of *Schistosoma japonicum*, tested the specific immune responses, and evaluated the protective efficacy through challenge infection in mice.

**Methods:**

DNA vaccine (pcDNA3.1-SjTPI.opt), adenoviral vectored vaccine (rAdV-SjTPI.opt), and recombinant protein vaccine (rSjTPI) were prepared and three types of heterologous prime-boost combinations, including DNA i.m. priming-rAdV i.m. boosting, rAdV i.m. priming-rAdV s.c. boosting, and rAdV i.m. priming-rSjTPI boosting strategies, were carried out. The specific immune responses and protective efficacies were evaluated in BALB/c mice

**Results:**

Results show that different immune profiles and various levels of protective efficacy were elicited by using different heterologous prime-boost combinations. A synergistic effect was observed using the DNA i.m. priming-rAdV i.m. boosting strategy; however, its protective efficacy was similar to that of rAdV i.m. immunization. Conversely, an antagonistic effect was generated by using the rAd i.m. priming-s.c. boosting strategy. However, the strategy, with rAdV i.m. priming- rSjTPI s.c. boosting, generated the most optimal protective efficacy and worm or egg reduction rate reaching up to 70% in a mouse model.

**Conclusions:**

A suitable immunization strategy, rAdV i.m. priming-rSjTPI boosting strategy, was developed, which elicits a high level of protective efficacy against *Schistosoma japonicum* infection in mice.

**Electronic supplementary material:**

The online version of this article (doi:10.1186/s13071-017-2036-5) contains supplementary material, which is available to authorized users.

## Background

Schistosomiasis is an important neglected tropical disease caused by trematode flatworms of the genus *Schistosoma* [1, 2]. Schistosomiasis transmission has been reported in 78 countries or regions in Africa, Asia and Southern America, and it has been estimated that at least 258.9 million people required preventive treatment in 2014 [3]. In China, schistosomiasis (caused by *S. japonicum*) is the most severe disease in history. Although extensive achievements have been made through its efficient control in the past several years, schistosomiasis remains endemic in the lowland marsh areas or lake regions of Hunan, Hubei, Jiangxi, Anhui and Jiangsu provinces and in the mountain areas of Sichuan and Yunnan provinces [4, 5]. In 2014, it was reported that there were 115,614 cases of schistosomiasis japonica distributed in 453 counties and 919,579 cattle raised in epidemic areas [6].

Praziquantel, an effective chemotherapy drug against *S. japonicum* that is relatively safe and of low cost, does not prevent host reinfection, and repeated chemotherapy treatment may generate drug resistance or decreased effectiveness against worms [7–10]. In China, schistosomiasis japonica is also a predominant zoonotic disease, and there are more than 40 animal reservoir hosts in the environment, including water buffalo, cattle, pigs and goats, which in turn largely contribute to sustaining the infection [11, 12]. Therefore, development of transmission-blocking veterinary vaccines is urgently needed for the prevention and efficient control of schistosomiasis in China.

Results from seroepidemiological investigation and studies of the radiation-attenuated cercariae model have provided evidence for the feasibility of vaccine development against schistosome infection [13, 14]. The World Health Organization (WHO) proposed that a vaccine with partial protective efficacy (≥ 50%) could ease host damage, reduce environmental pollution by eggs, and decrease overall morbidity [15]. Vaccines against *S. japonicum* have been studied for several years, and numerous antigen candidates from all life stages have been tested, including the 23-kDa membrane protein (Sj23), fatty acid-binding protein (SjFABP), and glutathione-S-transferase (SjGST). However, the protective efficacy induced by these antigens are not as ideal as expected [16–19]. Therefore, strategies for the improvement of protective efficacy should be further investigated for the development of novel vaccines against *S. japonicum* infection.

In recent years, a novel vaccination strategy, heterologous prime-boost, which uses unmatched vaccine delivery methods for immunization while using the same antigen, has been extensively applied in vaccine studies and has been determined to be more effective than traditional vaccination strategy of homologous prime-boost strategy [20]. Different prime-boost formats have been widely used in vaccine research against malaria, tuberculosis and AIDS, such as DNA priming-protein boosting and DNA priming-viral vectored vaccine boosting [21–23]. In our previous study, we cloned and optimized codon usage of the gene, triosephosphate isomerase of *S. japonicum* (SjTPI) for the first time [24]. Different types of vaccines were constructed, including DNA vaccine (pcDNA3.1-SjTPI, pcDNA3.1-SjTPI.opt), recombinant protein vaccine (rSjTPI), and recombinant adenoviral vaccine (rAdV-SjTPI.opt), and its protective efficacy was evaluated in a mouse model by using homologous prime-boost strategy. The results showed that worm reduction rates did not stabilize at the 50% level, a value recommended by the WHO. However, worm reduction rates significantly increased from 26.9 to 36.9% when a DNA priming-protein boosting strategy was used [17, 24–26].

To further improve protective efficacy, the present study immunized mice with three different types of heterologous prime-boost strategies based on our previously constructed vaccines, tested the specific immune responses, and evaluated the protective efficacy through challenge infection of *S. japonicum* with cercariae.

## Methods

### Animals and parasites

Six-week-old female BALB/c mice were purchased from the Shanghai Laboratory Animal Center (SLAC; Shanghai, China) and used in the vaccination studies. A Chinese mainland strain of *S. japonicum* infected *Oncomelania hupensis* was provided by Jiangsu Institute of Parasitic Diseases (Wuxi, China). Cercariae were collected from infected snails and used in animal challenges.

### Vaccine preparation

DNA vaccines (pcDNA3.1-SjTPI.opt) were previously constructed and purified by using Qiagen Plasmid Mega Kit (Qiagen, Dusseldorf, Germany) according to the manufacturer’s instructions. The final plasmid DNAs were in 0.01 M phosphate buffered solution (PBS) and verified for immunization by restriction enzyme digestion and DNA sequencing [24]. Recombinant proteins (rSjTPI) were purified from a prokaryotic expression system (pGEX-4T-3 as a vector, previously constructed), using a GST-tag purification modules (GE Healthcare; Buckinghamshire, UK), and thrombin (Sigma-Aldrich; St. Louis, USA) was used to remove the GST-tag [27]. The rSjTPI was diluted with PBS to a final concentration of 0.1 mg/ml, stored in aliquots at -80 °C and emulsified with an equal volume of Freund’s incomplete adjuvant (Sigma-Aldrich; St. Louis, USA) before immunization. Recombinant adenoviral vectored vaccines (rAdV-SjTPI.opt) were constructed and purified previously [26], stored in aliquots at -130 °C until use.

### Animal grouping and immunization

Mice were randomly divided into 11 different groups (16 mice in each group), which included a blank control (Control, without any immunization); pcDNA3.1 (DNA vector, immunized intramuscularly, i.m.); Ad vector (Ad Vector, immunized subcutaneously, s.c.); Ad vector (Ad Vector, i.m.); pcDNA-SjTPI.opt (DNA i.m.); rAdV-SjTPI.opt (rAdV s.c.); rAdV-SjTPI.opt (rAdV i.m.); rSjTPI (rSjTPI s.c.); pcDNA3.1-SjTPI.opt i.m. priming-rAdV-SjTPI.opt i.m. boosting (DNA i.m. + rAdV i.m.); rAdV-SjTPI.opt i.m. priming-rAdV-SjTPI.opt s.c. boosting [rAdV (i.m. + s.c.)]; and rAdV-SjTPI.opt i.m. priming-rSjTPI s.c. boosting (rAd i.m. + rSjTPI s.c.). Immunization was performed four times for the heterologous prime-boost groups (three times for priming and one for boosting) and three times for the other groups. The immunization doses for each vaccine were performed according to our previous studies. Briefly, the doses were 100 μg (DNA plasmids), 100 μg (rSjTPI) and 10^8^ pfu (rAdV) for each mouse in every immunization [24–26].

### Measurement of rSjTPI-specific antibody responses

Serum samples of each mouse were collected from caudal veins before immunization and challenge. Indirect enzyme linked immunosorbent assays (ELISAs) were used to measure rSjTPI-specific antibody responses, including IgG levels, IgG subclass (IgG1 and IgG2a) levels, IgG avidity, and IgG titer. rSjTPI (rTPI, purified previously) was used as the antigen source. To measure IgG, IgG1, and IgG2a levels, serum samples at a 1:100 dilution were added into ELISA plates (Nunc) that were coated with rTPI (0.2 μg/well) and recognized by second antibodies (HRP-conjugated goat-anti-mouse IgG, IgG1, and IgG2a, SouthernBiotech; Birmingham, USA) at a 1:5000 dilution. The optical density (OD) was read at a wavelength of 450 nm with a microplate reader (Antobio; Zhengzhou, China). To assess IgG avidity, an additional washing step with 6 M urea in PBST was performed after serum incubation to discard low avidity IgG, and the avidity index was calculated as the ratio of the OD_450_ treated and OD_450_ untreated, as described elsewhere [28, 29]. To measure IgG titers, serum samples from each mouse were examined using multiple dilutions (from 1:50 to 1:638,400) and the IgG titer was determined by comparing these to the OD_450_ value of the control (cut-off value ≥ 2.1 × the mean OD_450_ value of the control).

### Cytokine measurements

Two days before challenge, four mice from each group were randomly sacrificed, and cell suspensions were prepared under aseptic conditions by grinding the spleens and filtering through 200-mesh screens. The splenocytes from each mouse were cultured in triplicate (cell density: 5 × 10^5^ cells per well) in 96-well plates (Corning; NY, USA), incubated in RPMI 1640 medium (Hyclone; South Lagan, USA) supplemented with 10% fetal calf serum (Gibco; Grand Island, USA), and stimulated with rTPI (10 μg/ml), ConA (Sigma-Aldrich; St. Louis, USA, 10 μg/ml), or medium alone (mock) at 37 °C with 5% CO_2_ for 72 h. The supernatants were collected, and cytokine levels were measured using a BD Cytometric Bead Array (CBA) Mouse Th1/Th2/Th17 Cytokine Kit, according to the manufacturer’s protocols.

### Elispot assay

Cell suspensions from each group were prepared and stimulated as earlier described. The number of IL-4 and IFN-γ secreting cells were determined using mouse IL-4 and IFN-γ ELISpot kits (R&D; Minneapolis, USA), according to the manufacturer’s protocols. Spot forming units (SFU) were counted using the ELISpot ImmunoSpot S5 Analyzer (C.T.L., Germany) and analyzed using the C.T.L. ImmunoSpot image software version 5.1. The results were expressed as SFU for 1 × 10^6^ cells.

### Detection of specific antibodies against adenoviruses

Viral particles (VPS) of adenoviruses were determined by using the OD_260_ method (1 OD_260_ = 1.1 × 10^12^ VPS/ml) [30]. In addition, indirect ELISAs were performed to detect adenovirus-specific antibody levels. Adenoviruses were used as the antigen source. Serum samples from each group at a 1:100 dilution were added into plates coated with adenovirus (10^7^ VPS/well) and recognized by secondary antibodies (HRP-conjugated goat anti-mouse IgG, SouthernBiotech; Birmingham, USA) at a 1:5000 dilution. ODs were read at a wavelength of 450 nm using a microplate reader (Antobio; Zhengzhou, China).

### Animal challenge and efficacy observation

Two weeks after the last immunization, each mouse was challenged with 40 ± 1 *S. japonicum* cercariae by abdominal skin penetration. Forty-two days post-challenge, all mice were sacrificed and perfused to observe worm burdens. Worm (female worm) reduction rate was calculated by using the following formula: Reduction rate (%) = [1 - Average total worm (or female worm) burden in each group/Average total worm (or female worm) burden in the control group] × 100. Whole livers from each mouse were collected, weighted, and digested with 5 ml of 5% potassium hydroxide (KOH) at 37 °C for 72 h. Ten microliters of the liver digest were loaded onto a glass counting slide to count the number of eggs (repeated 3 times), and the number of eggs per gram liver from each mouse was calculated. Liver egg reduction rates were calculated by using the following formula: Reduction rate (%) = (1 - Average number of eggs per gram liver in each group/Average number of eggs per gram liver in the control group) × 100.

### Histopathological examination of livers

Areas of single egg granuloma in the livers were observed by using sectioned liver tissues (1–5 cm^3^) collected from each mouse. The procedures of section preparation were according to standard histological operations, including fixation in 4% formaldehyde, dehydration in alcohol, embedding in paraffin, and staining with hematoxylin-eosin. Egg granulomas in the liver were observed and imaged under a light microscope (Olympus BX51; Tokyo, Japan). Areas of each single egg granuloma were determined using a computerized image analysis system (JD801 Version 1.0; Nanjing, China). Granuloma sizes were expressed as the means of areas measured in μm^2^ ± SD.

### Statistical analysis

Statistical analysis was performed using the SPSS software (Version 19.0). One-way ANOVA was used for data comparison among different groups, and the paired Student’s *t*-test was used to compare any two means. *P-*values < 0.05 or < 0.01 were considered statistically significant.

## Results

### Specific immune responses and protective efficacy induced through DNA i.m. priming-rAdV i.m. boosting strategy against *S. japonicum* infection

**Fig. 1:**
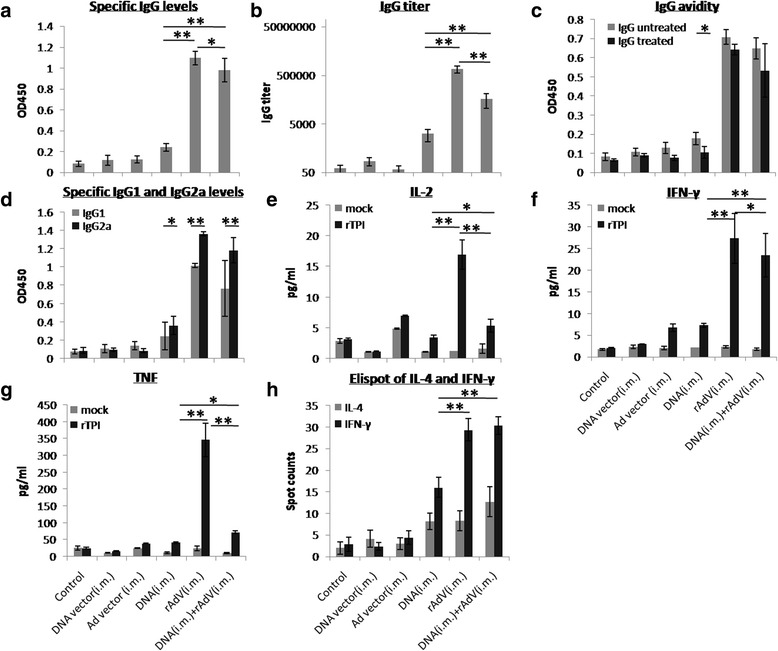
rSjTPI-specific immune responses induced by a DNA vector (i.m.), Ad vector (i.m.), DNA (i.m.), rAdV (i.m.), DNA (i.m.) + rAdV (i.m.) immunized groups and the control group. **a** IgG responses. **b** IgG titers. **c** IgG avidity. **d** IgG1 and IgG2a responses. **e** IL-2 levels. **f** IFN-γ levels. **g** TNF levels. **h** Spot counts of IL-4 and number of IFN-γ secreting cells. Each bar represents the mean ± standard deviation (SD). *P < 0.05; **P < 0.01

Compared to the control or vector immunized group, DNA i.m., rAdV i.m., and DNA i.m. + rAdV i.m. immunization induced significantly higher IgG (ANOVA, *F*_(5,42)_ = 135.76, *P* < 0.001), IgG1 (ANOVA, *F*_(5,42)_ = 33.99, *P* < 0.001), and IgG2a (ANOVA, *F*_(5,42)_ = 157.70, *P* < 0.001) levels and IgG titers (ANOVA, *F*_(5,42)_ = 78.33, *P* < 0.001), respectively. Levels of IgG and IgG titers induced by DNA i.m. + rAdV i.m. immunization were significantly higher compared to that induced by DNA i.m. immunization (*t*-test, *t*_(15)_ = 15.93, *P* < 0.001 and, *t*_(15)_ = 3.24, *P* = 0.005), but significantly lower compared to that induced by rAdV i.m. immunization (*t*-test, *t*_(15)_ = 5.52, *P* = 0.02 and *t*_(15)_ = 3.98, *P* = 0.001) (Fig. 1a, b). rAdV i.m. and DNA i.m. + rAdV i.m. immunization elicited higher IgG avidity when compared to that induced by DNA i.m. immunization, and IgG avidity indices were 0.909, 0.823, and 0.597, respectively (*t*-test, *t*_(15)_ = 5.89, *P* < 0.001 and *t*_(15)_ = 8.21, P < 0.001) (Fig. 1c). DNA i.m., rAdV i.m., and DNA i.m. + rAdV i.m. immunization induced similar IgG2a biased levels, and IgG2a/IgG1 ratios were 1.47, 1.34, and 1.55, respectively. However, the highest IgG2a levels were produced by rAdV i.m. immunization (ANOVA, *F*_(2,21)_ = 66.22, *P* < 0.001) (Fig. 1d).

CBA and ELISpot analysis showed that splenocytes from DNA i.m., rAdV i.m., and DNA i.m. + rAdV i.m. immunized groups produced higher levels of Th1 cytokines (IL-2, IFN-γ, and TNF) than those immunized with a vector (ANOVA, *F*_(5,42)_ = 7.17, *P* < 0.001; *F*_(5,42)_ = 9.27, *P* < 0.001; *F*_(5,42)_ = 16.18, *P* < 0.001, respectively). Cytokine levels (IL-2, IFN-γ, and TNF) induced by DNA i.m. + rAdV i.m. immunization were higher than those induced by DNA i.m. immunization (*t*-test, *t*_(15)_ = 2.60, *P* = 0.02 for IL-2 in DNA i.m. + rAdV i.m. *vs* DNA i.m.; *t*_(15)_ = 4.07, *P* = 0.001 for IFN-γ in DNA i.m. + rAdV i.m. *vs* DNA i.m.; *t*_(15)_ = 2.73, *P* = 0.03 for TNF in DNA i.m. + rAdV i.m. *vs* DNA i.m.), but lower than that induced by rAdV i.m. immunization (*t*-test, *t*_(15)_ = 4.87, *P* < 0.001 for IL-2 in DNA i.m. + rAdV i.m. *vs* rAdV i.m.; *t*_(15)_ = 2.95, *P* = 0.02 for IFN-γ in DNA i.m. + rAdV i.m. *vs* rAdV i.m.; *t*_(15)_ = 5.27, *P* < 0.001 for TNF in DNA i.m. + rAdV i.m. *vs* rAdV i.m.). However, no significant differences in the amount of IFN-γ secreting cells between rAdV i.m. and DNA i.m. + rAdV i.m. immunizations were observed (*t*-test, *t*_(19)_ = 1.73, *P* = 0.10) (Fig. 1e-h). Various types of Th2 (IL-4, IL-6, and IL-10) and Th17 (IL-17A) cytokines were detected at low levels (Additional file 1: Figure S1).

The results of protective efficacy are shown in Fig. 5 and Table 1. Compared to the control and vector groups, DNA i.m., rAdV i.m., and DNA i.m. + rAdV i.m. immunizations produced lower numbers of adult worms, female worms, eggs in the liver (ANOVA, *F*_(5,64)_ = 22.57, *P* < 0.001; *F*_(5,64)_ = 32.87, *P* < 0.001; *F*_(5,64)_ = 29.35, *P* < 0.001, respectively, see Table 1), and smaller areas of single-egg granuloma in the liver (ANOVA, *F*_(5,64)_ = 39.25, *P* < 0.001, see Fig. 5). DNA i.m. + rAdV i.m. immunization produced lower numbers of adult worms, female worms, eggs in the liver, and smaller areas of single-egg granuloma in the liver compared to that produced by DNA i.m. immunization (*t*-test, *t*_(22)_ = 6.57, *P* < 0.001; *t*_(22)_ = 3.68, *P* = 0.001; *t*_(22)_ = 3.57, *P* = 0.002; *t*_(22)_ = 8.29, *P* < 0.001, respectively). However, no statistically significant differences in protective efficacies between DNA i.m. + rAdV i.m. and rAdV i.m. immunizations were observed (*t*-test, *t*_(22)_ = 2.02, *P* = 0.055; *t*_(22)_ = 1.72, *P* = 0.10; *t*_(22)_ = 1.57, *P* = 0.15; *t*_(22)_ = 1.14, *P* = 0.31, respectively).

**Table 1:** Summary of the protective efficacies of the different immunization groups

Group	No. of mice	Adult worms		Female worms		Eggs in the liver	
		No. of worms	Reduction (%)	No. of worms	Reduction (%)	No. of eggs	Reduction (%)
Control	11	28 .33 ± 2.55	–	13.67 ± 1.50	–	114,434 ± 17,170	–
DNA vector (i.m.)	12	27.13 ± 6.42	4.26	13.25 ± 3.15	3.05	107,435 ± 25,289	6.12
Ad vector (i.m.)	12	26.50 ± 3.16	6.47	13.00 ± 1.77	4.88	106,826 ± 18,808	6.65
Ad vector (s.c.)	11	26.27 ± 2.72	7.27	12.73 ± 1.35	6.87	109,061 ± 25,571	4.70
DNA (i.m.)	12	19 .22 ± 1.64^a^	32.16	9.33 ± 1.00^a^	31.71	72,947 ± 26,998^a^	36.25
rAdV (i.m.)	12	14.00 ± 4.84^a,b^	50.59	6.18 ± 1.94^a,b^	54.77	54,883 ± 26,892^a,b^	52.04
rAdV (s.c.)	11	18.00 ± 5.24^a^	36.47	8.63 ± 2.83^a^	36.89	67,077 ± 21,277^a^	41.38
rSjTPI (s.c.)	12	20.78 ± 4.52^a^	26.67	10.22 ± 2.39^a^	25.20	70,993 ± 28,772^a^	37.96
DNA (i.m.) + rAdV (i.m.)	11	15.55 ± 4.61^a,c^	45.13	6.82 ± 2.71^a,c^	50.11	51,991 ± 11,395^a,c^	54.57
rAdV (i.m. + s.c.)	12	15.75 ± 6.09^a,d^	44.41	7.58 ± 2.81^a,d^	44.51	49,095 ± 14,323^a,d^	57.10
rAdV (i.m.) + rSjTPI (s.c.)	12	7.91 ± 2.47^a,e^	72.09	3.73 ± 1.19^a,e^	72.73	31,891 ± 17,776^a,e^	72.13

^a^Statistically significant differences (*P* < 0.01), compared to the control or vector control group

^b^Statistically significant differences (*P* < 0.01), compared to the DNA (i.m.), rAdV (s.c.), or rSjTPI (s.c.) group

^c^Statistically significant difference (*P* < 0.01), compared to the DNA (i.m.) group

^d^Statistically significant difference (*P* < 0.05), compared to the rAdV (s.c.) group

^e^Statistically significant differences (*P* < 0.01), compared to each group

### Specific immune responses and protective efficacy induced by rAdV i.m. priming-rAdV s.c. boosting strategy against *S. japonicum* infection

**Fig. 2:**
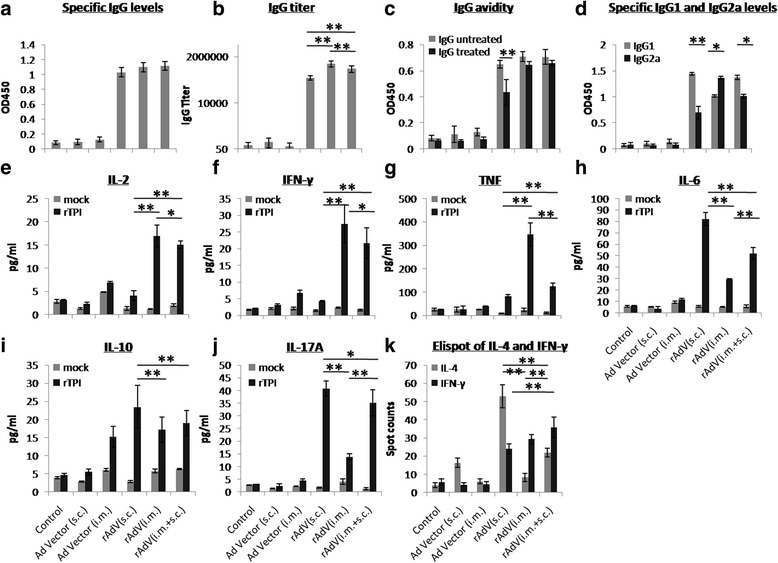
rSjTPI-specific immune responses induced by Ad vector (s.c.), Ad vector (i.m.), rAdV (s.c.), rAdV (i.m.), and rAdV (i.m. + s.c.) immunized groups and the control group. **a** IgG responses. **b** IgG titers. **c** IgG avidity. **d** IgG1 and IgG2a responses. **e** IL-2 levels. **f** IFN-γ levels. **g** TNF levels. **h** IL-6 levels. **i** IL-10 levels. **j** IL-17A levels. **k** Spot counts of IL-4 and number of IFN-γ secreting cells. Each bar represents the mean ± standard deviation (SD). *P < 0.05; **P < 0.01

Compared to the control or vector immunized group, rAdV s.c., rAdV i.m., and rAdV (i.m. + s.c.) immunization induced significantly higher IgG, IgG1, and IgG2a levels and IgG titers, respectively (ANOVA, *F*_(5,42)_ = 237.76, *P* < 0.001; *F*_(5,42)_ = 99.21, *P* < 0.001; *F*_(5,42)_ = 109.38, *P* < 0.001; and  *F*_(5,42)_ = 119.36, *P* < 0.001, respectively). The IgG and IgG titers induced by rAdV (i.m. + s.c.) immunization were significantly elevated compared to that induced by rAdV s.c. immunization (*t*-test, *t*_(15)_ = 2.61, *P* = 0.02 and *t*-test, *t*_(15)_ = 13.14, *P* < 0.001), but IgG titers were significantly lower to that induced by rAdV i.m. immunization (*t*-test, *t*_(15)_ = 18.03, *P* < 0.001) (Fig. 2a, b). rAdV i.m. and rAdV (i.m. + s.c.) immunizations elicited higher IgG avidity compared to that induced by rAdV s.c. immunization, and IgG avidity indices were 0.909, 0.903, and 0.480, respectively (see Fig. 2c). rAdV s.c. and rAdV (i.m. + s.c.) immunization induced similar IgG1 biased levels, and the IgG2a/IgG1 ratio was 1.34 and 0.74, respectively. However, the highest IgG1 levels were produced by rAdV s.c. immunization (ANOVA, *F*_(2,21)_ =4.71, *P* = 0.03, Fig. 2d).

CBA and ELISpot analysis showed that splenocytes from rAdV s.c., rAdV i.m., and rAdV (i.m. + s.c.) immunized groups produced higher levels of cytokines (IL-2, IFN-γ, TNF, IL-6, IL-10, and IL-17A) or number of IL-4/IFN-γ secreting cells than that immunized with a vector (ANOVA, *F*_(5,42)_ = 21.17, *P* < 0.001; *F*_(5,42)_ = 9.82, *P* < 0.001; *F*_(5,42)_ = 18.12, *P* < 0.001; *F*_(5,42)_ = 17.07, *P* < 0.001; *F*_(5,42)_ = 4.37, *P* = 0.005; *F*_(5,42)_ = 9.77, *P* < 0.001; *F*_(5,54)_ = 6.18, *P* < 0.001, respectively). Th2-biased cytokine expression profiles were produced through rAdV s.c. immunization, whereas rAdV i.m. immunization generated Th1-biased cytokine expression profiles. CBA analysis indicated that Th1 type cytokine (IL-2, IFN-γ, and TNF) levels produced by rAdV (i.m. + s.c.) immunization were lower than that induced by rAdV i.m. immunization (*t*-test, *t*_(15)_ = 2.29, *P* = 0.03; *t*_(15)_ = 2.61, *P* = 0.02; *t*_(15)_ = 4.07, *P* = 0.001, respectively), and Th2/17 type cytokine (IL-6, IL-10, and IL-17A) levels were also lower than that induced by rAdV s.c. immunization (*t*-test, *t*_(15)_ = 3.73, *P* = 0.002; *t*_(15)_ = 3.29, *P* = 0.005; *t*_(15)_ = 2.95, *P* = 0.01, respectively) (Fig. 2e-j). No IL-4 was detected by CBA (data not shown). ELISpot analysis showed that the number of IL-4 secreting cells induced by rAdV (i.m. + s.c.) was lower than that induced by rAdV s.c. (*t*-test, *t*_(19)_ = 3.58, *P* = 0.002), whereas the amount of IFN-γ secreting cells did not significantly differ from that induced by rAdV i.m. immunization (Fig. 2k).

The results of the assessment of protective efficacy are shown in Fig. 5 and Table 1. Compared to the control and vector groups, rAdV s.c., rAdV i.m., and rAdV (i.m. + s.c.) immunizations produced lower numbers of adult worms, female worms, eggs in the liver (ANOVA, *F*_(5,63)_ = 52.31, *P* < 0.001; *F*_(5,63)_ = 33.28, *P* < 0.001; *F*_(5, 63)_ = 29.76, *P* < 0.001, respectively, see Table 1), and smaller areas of single-egg granulomas in the liver (ANOVA, *F*_(5,63)_ = 30.11, *P* < 0.001, Fig. 5). rAdV (i.m. + s.c.) immunization produced a lower number of adult worms, female worms, eggs in the liver, and smaller areas of single-egg granulomas in the liver compared to that produced by rAdV s.c. immunization(*t*-test, *t*_(22)_ = 2.34, *P* = 0.04; *t*_(22)_ = 2.81, *P* = 0.01; *t*_(22)_ = 2.27, *P* = 0.04; *t*_(22)_ = 2.77, *P* = 0.015, respectively). However, no statistically significant differences in protective parameters between rAdV (i.m. + s.c.) and rAdV i.m. immunizations were observed.

### Specific immune responses and protective efficacy induced by rAdV i.m. priming-rSjTPI s.c. boosting strategy against *S. japonicum* infection

**Fig. 3:**
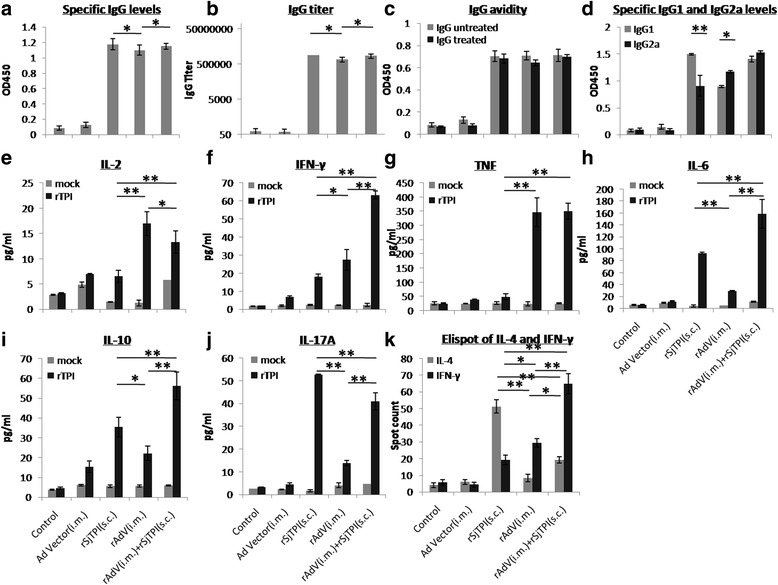
rSjTPI-specific immune responses induced by an Ad vector (i.m.), rSjTPI (s.c.), rAdV (i.m.), and rAdV (i.m.) + rSjTPI (s.c.) immunized groups and the control group. **a** IgG responses. **b** IgG titers. **c** IgG avidity. **d** IgG1 and IgG2a responses. **e** IL-2 levels. **f** IFN-γ levels. **g** TNF levels. **h** IL-6 levels. **i** IL-10 levels. **j** IL-17A levels. **k** Spot counts of IL-4 and number of IFN-γ secreting cells. Each bar represents the mean ± standard deviation (SD). *P < 0.05; **P < 0.01

Compared to the control group, rAdV i.m., rSjTPI s.c., and rAdV i.m. + rSjTPI s.c. immunizations elicited higher IgG levels and IgG titers (ANOVA, *F*_(4,35)_ = 137.21, *P* < 0.001; *F*_(4,35)_ = 39.31, *P* < 0.001, respectively), whereas rSjTPI s.c. and rAdV i.m. + rSjTPI s.c. immunizations elicited higher IgG responses (including IgG levels and IgG titers) than that using rAdV i.m. immunization (*t*-test, *t*_(15)_ = 2.21, *P* = 0.04 for IgG in rSjTPI s.c. *vs* rAdV i.m.; *t*_(15)_ = 2.60, *P* = 0.02 for IgG titers in rSjTPI s.c. *vs* rAdV i.m.; *t*_(15)_ = 2.37, *P* < 0.034 for IgG in rAdV i.m. + rSjTPI s.c. *vs* rAdV i.m.; *t*_(15)_ = 2.92, *P* = 0.01 for IgG titers in rAdV i.m. + rSjTPI s.c. *vs* rAdV i.m., respectively) (Fig. 3a, b). The IgG avidity indices of the three groups were 0.973, 0.809, and 0.983, respectively (Fig. 3c). The three different immunization types elicited various levels of IgG subclasses (Fig. 3d). rSjTPI s.c. immunization induced a higher IgG1 level, with a IgG2a/IgG1 ratio of 0.61(*t*-test, *t*_(15)_ = 5.01, *P* < 0.001). rAdV i.m. immunization induced a higher IgG2a level, with a IgG2a/IgG1 ratio of 1.31(*t*-test, *t*_(15)_ = 2.95, *P* = 0.01). The IgG1 and IgG2a levels were simultaneously elicited by rAdV i.m. + rSjTPI s.c. immunization, with an IgG2a/IgG1 ratio of 1.08. Furthermore, rAdV i.m. priming-rSjTPI s.c. boosting immunization elicited the highest specific IgG2a levels (ANOVA, *F*_(2,21)_ = 37.21, *P* < 0.001).

CBA and ELISpot analyses showed that splenocytes from rSjTPI s.c., rAdV i.m. and rAdV i.m. + rSjTPI s.c. immunized groups produced higher levels of cytokines (IL-2, IFN-γ, TNF, IL-6, IL-10, and IL-17A) or numbers of IL-4/IFN-γ secreting cells than that immunized with a vector or the control group (ANOVA, *F*_(4,35)_ = 41.25, *P* < 0.001; *F*_(4,35)_ = 10.07, *P* < 0.001; *F*_(4,35)_ = 28.34, *P* < 0.001; *F*_(4,35)_ = 25.19, *P* < 0.001; *F*_(4,35)_ = 14.24, *P* < 0.005; *F*_(4,35)_ = 31.17, *P* < 0.001; *F*_(4,45)_ = 16.46, *P* < 0.001, respectively) (Fig. 3e-k). Splenocytes from mice that underwent rAdV i.m. immunization produced higher levels of Th1 cytokines (IL-2, TNF, and IFN-γ), whereas rSjTPI s.c. immunization induced higher levels of Th2 (IL-4, IL-6, and IL-10) and Th17 (IL-17A) cytokines. Furthermore, the IFN-γ, IL-6, IL-10 levels and the number of IFN-γ secreting cells elicited by rAdV i.m. + rSjTPI s.c. immunization were higher than those generated using rAdV i.m.(*t*-test, *t*_(15)_ = 4.07, *P* = 0.001; *t*_(15)_ = 8.28, *P* < 0.001; *t*_(15)_ = 3.29, *P* = 0.005; *t*_(19)_ = 3.58, *P* = 0.002, respectively), or rSjTPI s.c. immunizations(*t*-test, *t*_(15)_ = 14.11, *P* < 0.001; *t*_(15)_ = 3.28, *P* = 0.005; *t*_(15)_ = 4.05, *P* = 0.001; *t*_(19)_ = 13.18, *P* < 0.001, respectively), and the IL-17A levels in the rAdV i.m. + rSjTPI s.c. immunization group were higher than that in the rAdV i.m. group but lower than that in the rSjTPI s.c. group(*t*-test, *t*_(15)_ = 19.22, *P* < 0.001; *t*_(15)_ = 3.68, *P* = 0.002, respectively). No IL-4 was detected by CBA (data not shown).

Figure 5 and Table 1 show the protective efficacy of various immunization groups. Compared to the control group and the Ad vector i.m. immunization group, rSjTPI s.c., rAdV i.m. and rAdV i.m. + rSjTPI s.c. immunizations resulted in lower numbers of adult worm, female worms, eggs in the liver (ANOVA, *F*_(4,54)_ = 11.37, *P* < 0.001; *F*_(4,54)_ = 23.57, *P* < 0.001; *F*_(4,54)_ = 26.19, *P* < 0.001, respectively, see Table 1), and smaller areas of single-egg granuloma in the liver (ANOVA, *F*_(4,54)_ = 10.05, *P* < 0.001, Fig. 5). rAdV i.m. immunization produced a lower number of adult worms, female worms, eggs in the liver, and smaller areas of single-egg granuloma in the liver compared to that produced by rSjTPI s.c. immunization (*t*-test, *t*_(23)_ = 3.77, *P* = 0.001; *t*_(23)_ = 4.21, *P* < 0.001; *t*_(23)_ = 3.49, *P* = 0.002; *t*_(23)_ = 8.58, *P* < 0.001, respectively). However, rAdV i.m. + rSjTPI s.c. immunization produced the lowest number of adult worms, female worms, eggs in the liver and smallest areas of single-egg granulomas in the liver compared to that induced in the other groups (*t*-test, *t*_(23)_ = 5.77, *P* < 0.001; *t*_(23)_ = 4.29, *P* < 0.001; *t*_(23)_ = 5.89, *P* < 0.001; *t*_(23)_ = 8.58, *P* < 0.001, in rAdV i.m. + rSjTPI s.c. *vs* rAdV i.m., respectively; *t*-test, *t*_(23)_ = 7.72, *P* < 0.001; *t*_(23)_ = 6.11, *P* < 0.001; *t*_(23)_ = 8.19, *P* < 0.001; *t*_(23)_ = 7.31, *P* < 0.001, in rAdV i.m. + rSjTPI s.c. *vs* rSjTPI s.c., respectively).

### Comparison of immune responses and protective efficacy induced by three heterologous prime-boost strategies

**Fig. 4:**
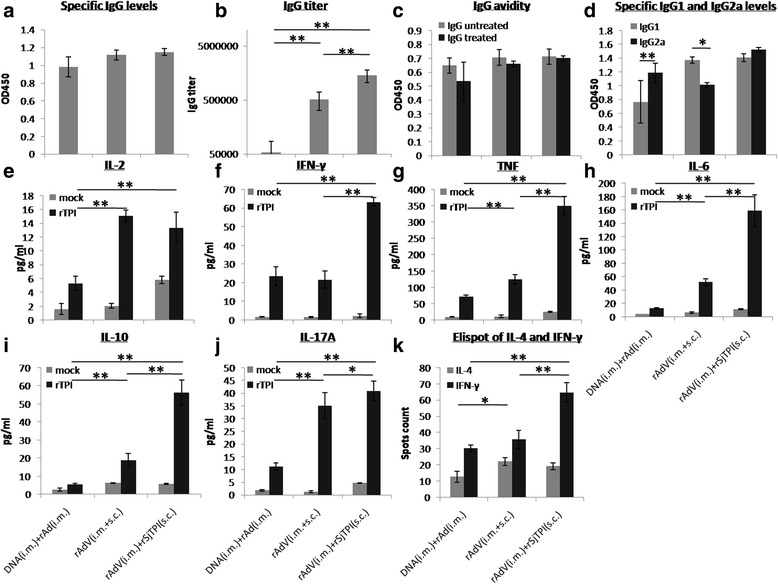
rSjTPI-specific immune responses induced by DNA (i.m.) + rAdV(i.m.), rAdV (i.m. + s.c.), and rAdV (i.m.) + rSjTPI (s.c.) immunized groups. **a** IgG responses. **b** IgG titers. **c** IgG avidity. **d** IgG1 and IgG2a responses. **e** IL-2 levels. **f** IFN-γ levels. **g** TNF levels. **h** IL-6 levels. **i** IL-10 levels. **j** IL-17A levels. **k** Spot counts of IL-4 and number of IFN-γ secreting cells. Each bar represents the mean ± standard deviation (SD). *P < 0.05; **P < 0.01

The specific immune responses induced by three heterologous prime-boost strategies are summarized in Fig. 4. The high IgG levels and IgG avidity were elicited by these strategies (Fig. 4a, c), although the IgG titers and the IgG subclasses differed (Fig. 4b, d). The rAdV i.m. priming-rSjTPI s.c. boosting strategy produced the highest IgG titers, whereas the DNA i.m. priming-rAdV s.c. boosting strategy produced the lowest (ANOVA, *F*_(2,22)_ = 11.27, *P* < 0.001) (Fig. 4b). A higher IgG2a level was elicited by DNA i.m. + rAdV i.m. immunization (*t*-test, *t*_(15)_ = 3.29, *P* = 0.005), whereas, a higher IgG1 level was elicited by rAdV (i.m. + s.c.) immunization (*t*-test, *t*_(15)_ = 2.61, *P* = 0.02). The IgG1 and IgG2a levels were simultaneously elicited in the rAdV i.m. + rSjTPI s.c. immunization group (Fig. 4d).

CBA and ELISpot analyses showed that rAdV i.m. + rSjTPI s.c. immunization produced the highest cytokine levels (IFN-γ, TNF, IL-6, IL-10, and IL-17A) and number of IFN-γ secreting cells (ANOVA, *F*_(2,22)_ = 9.72, *P* < 0.001; *F*_(2,22)_ = 8.27, *P* < 0.001; *F*_(2,22)_ = 11.25, *P* < 0.001; *F*_(2,22)_ = 18.01, *P* < 0.001; *F*_(2,22)_ = 19.38, *P* < 0.001; *F*_(2,27)_ = 22.01, *P* < 0.001, respectively), and no significant differences in IL-2 levels between rAdV (i.m. + s.c.) and rAdV i.m. + rSjTPI s.c. groups were observed (*t*-test, *t*_(15)_ = 1.75, *P* = 0.10). rAdV (i.m. + s.c.) immunization induced higher cytokine levels (IL-2, TNF, IL-6, IL-10, and IL-17A) and numbers of IL-4 secreting cells than that in the DNA i.m. + rAdV i.m. group (*t*-test, *t*_(15)_ = 3.74, *P* = 0.002; *t*_(15)_ = 5.35, *P* < 0.001; *t*_(15)_ = 6.31, *P* < 0.001; *t*_(15)_ = 5.89, *P* < 0.001; *t*_(19)_ = 2.54, *P* = 0.02, respectively). However, no significant differences in IFN-γ levels and number of IFN-γ secreting cells between these two groups were observed (*t*-test, *t*_(15)_ = 0.87, *P* = 0.40; *t*_(19)_ = 1.73, *P* = 0.10, respectively Fig. 4e-k).

**Fig. 5:**
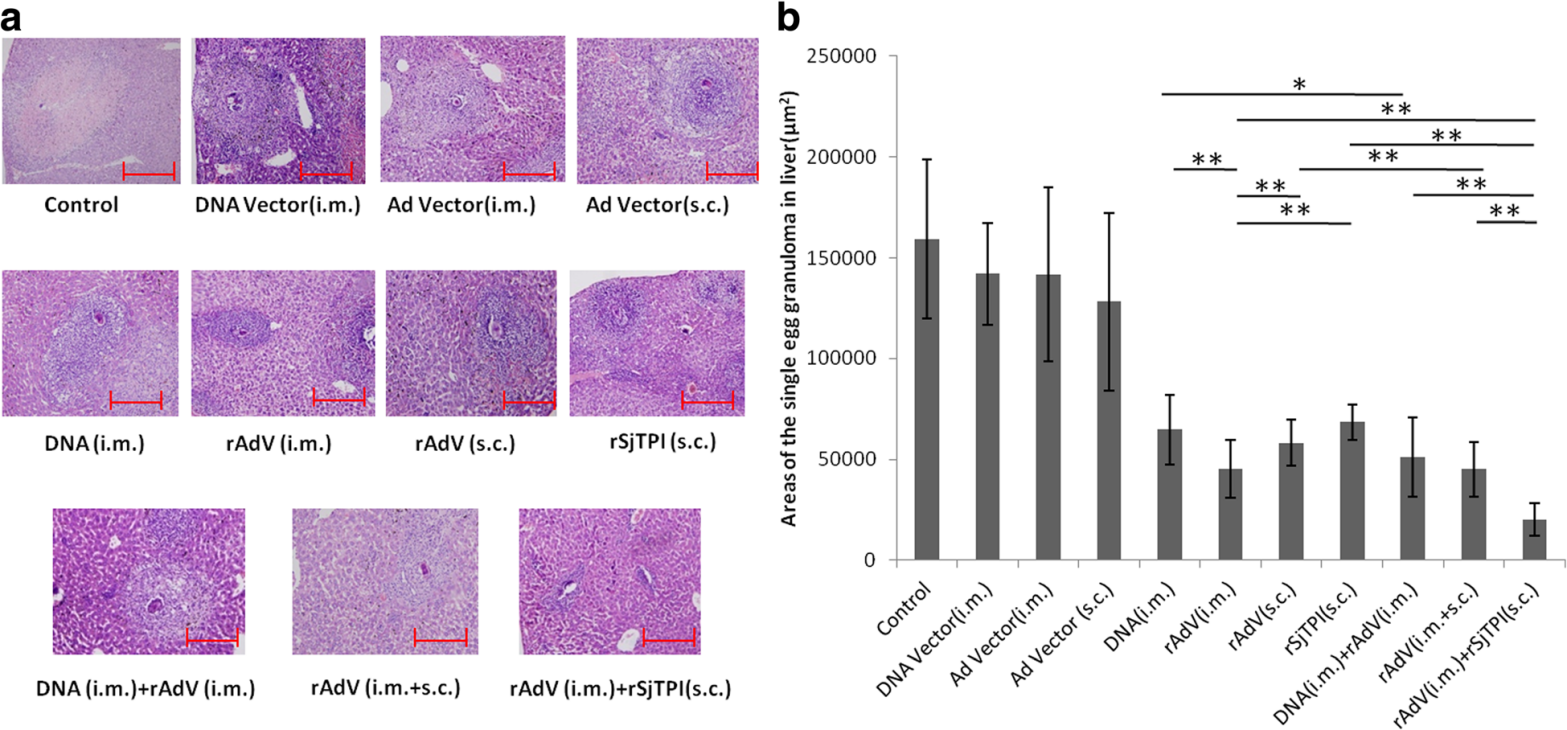
The single-egg granuloma responses in the liver induced by each immunization strategy. **a** Representative granuloma of each group induced by a single egg in liver (magnification factor 10 × 10; *Scale-bars*: 100 μm). **b** Areas of the single-egg granuloma in liver. Data are expressed as the mean ± standard deviation (SD). *P < 0.05; **P < 0.01

rAdV i.m. + rSjTPI s.c. immunization produced the lowest number of adult worms, female worms, eggs in liver, and smallest areas of single-egg granulomas in the liver compared to that induced in the other two groups (ANOVA, *F*_(2, 32)_ = 8.51, *P* < 0.001; *F*_(2,32)_ = 6.61, *P* < 0.001; *F*_(2,32)_ = 10.09, *P* < 0.001; *F*_(2,32)_ = 8.91, *P* < 0.001, respectively, Fig. 5 and Table 1). In addition, no significant differences in protective parameters between the DNA i.m. + rAdV i.m. and rAdV (i.m. + s.c.) groups were observed (*t*-test, *t*_(22)_ = 0.86, *P* = 0.40; *t*_(22)_ = 1.31, *P* = 0.20; *t*_(22)_ = 1.97, *P* = 0.06; *t*_(22)_ = 1.52, *P* = 0.15, respectively).

### Specific IgG levels against adenoviruses as induced by different groups

**Fig. 6:**
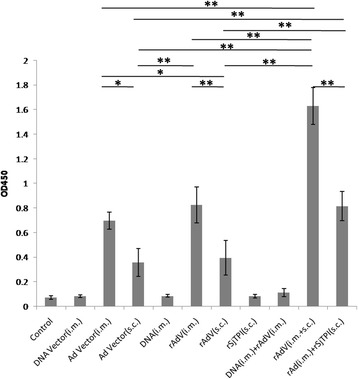
Adenovirus-specific IgG responses by immunized group. Each bar represents the mean ± standard deviation (SD). *P < 0.05; **P < 0.01

The specific IgG levels against adenoviruses are shown in Fig. 6. Compared to the control group, Ad vector i.m., Ad vector s.c., rAdV i.m., rAdV s.c., rAdV (i.m. + s.c.), and rAdV i.m. + rSjTPI s.c. immunization elicited higher IgG levels against adenoviruses (ANOVA, *F*_(10,77)_ = 8.32, *P* <0.001). On the other hand, DNA vector i.m., DNA i.m., rSjTPI s.c., and DNA i.m. + rAdV i.m. immunization did not elicit specific IgG levels against adenoviruses. Adenoviruses (Ad vector or rAdV) immunized intramuscularly elicited higher specific IgG levels than that induced by subcutaneous immunization (*t*-test, *t*_(15)_ = 3.29, *P* = 0.005 for IgG in rAdV i.m. *vs* rAdV s.c.; *t*_(15)_ = 2.61, *P* = 0.02 for IgG in Ad Vector i.m. *vs* Ad Vector s.c.). Furthermore, rAdV (i.m. + s.c.) immunization elicited the highest adenovirus specific IgG levels (*t*-test, *t*_(15)_ = 5.31, *P* < 0.001 for IgG in rAdV i.m. + rAdV s.c. *vs* Ad Vector i.m.; *t*_(15)_ = 7.42, *P* < 0.001 for IgG in rAdV i.m. + rAdV s.c. *vs* rAdV i.m.; *t*_(15)_ = 8.51, *P* < 0.001 for IgG in rAdV i.m. + rAdV s.c. *vs* rAdV s.c.; *t*_(15)_ = 7.62, *P* < 0.001 for IgG in rAdV i.m. + rAdV s.c. *vs* rAdV i.m. + rSjTPI s.c.).

## Discussion

We evaluated specific immune responses and protective efficacy against *S. japonicum* in mice using three types of heterologous prime-boost combinations, including DNA i.m. priming-rAdV i.m. boosting, rAdV i.m. priming-rAdV s.c. boosting, and rAdV i.m. priming-rSjTPI boosting strategies. The results of the present study showed that various heterologous prime-boost combinations elicit different immune profiles, and different levels of protective efficacy were generated accordingly. However, the strategy, priming with rAdV intramuscularly, and boosting with rSjTPI subcutaneously, generated the optimal protective efficacy and the worm or egg reduction rate reaching up to 70% in a mouse model.

Previous studies have clearly shown that heterologous prime-boost vaccination elevates protective efficacy [20–23]. However, its underlying mechanism has not been clearly elucidated. Different vaccine vectors or delivery systems may deliver and present protective antigens in their own way, and this may stimulate the host immune systems to generate antibodies with higher avidity, a broad spectrum of specific immune responses, and the circumvention of anti-vector effects [31, 32]. Furthermore, previous studies have shown that a high level of specific Th1 (IFN-γ and IgG2a) responses is associated with a high degree of protection against *S. japonicum* infection in animal models [33, 34]. However, specific Th2 responses may also contribute to protection [35]. In our previous studies, a series of vaccines based on triosephosphate isomerase of *S. japonicum* (SjTPI) were constructed, including a DNA vaccine (pcDNA3.1-SjTPI.opt), a protein vaccine (rSjTPI), and an adenoviral vectored vaccine (rAdV-SjTPI.opt). Animal experiments have shown that DNA and adenoviral vectored vaccines elicit a specific Th1-biased immune response when immunized intramuscularly, whereas, protein and adenoviral vectored vaccines elicit a specific Th2-biased immune response when immunized subcutaneously [19, 20, 25, 26]. To obtain a vaccination strategy with higher efficacy against *S. japonicum*, we designed three types of heterologous prime-boost strategies in the present study.

DNA and adenoviral vaccines could express the antigen in muscular cells when immunized intramuscularly, which present protective antigens through the MHC-I processing pathway and could elicit specific Th1-biased immune responses [36, 37]. Furthermore, the anti-vector effect might be minimized using different vaccine delivery systems (vectors). In the present study, a synergistic effect was produced by using a DNA i.m. priming-rAdV i.m. boosting strategy as indicated by an enhancement of Th1-biased immune responses and protective efficacy compared to that using DNA i.m. immunization. However, the observed protective efficacy was similar to that with rAdV i.m. immunization. This may be due to differences in vivo transfection efficiency between DNA plasmids and adenoviruses immunized intramuscularly because DNA plasmids passively enter cells (penetrate), whereas adenoviruses enter cells through active infection (transfection) [36, 37]. This difference may affect the expression of the delivered antigens and ultimately lead to differences in specific immune responses and protective efficacies accordingly.

Replication-deficient adenoviral vectors retain its actively invading ability to target cells and show high transfection efficiency when applied to a vaccine delivery systems [38, 39]. Previous studies have shown that different types of immune responses and various levels of protective efficacy are elicited when immunized intramuscularly or subcutaneously [26]. To gain a broad spectrum of specific immune responses, we exploited rAdV-SjTPI.opt immunized intramuscularly as the priming vaccine and used different boosting vaccines (rAdV-SjTPI.opt or rSjTPI, all immunized subcutaneously). Different outcomes were obtained using these two heterologous prime-boost strategies. A synergistic effect was produced by the rAdV i.m. priming-rSjTPI boosting strategy, as indicated by the board spectrum of immune responses and high protective efficacy (> 70%) against infection. However, an antagonistic effect was produced by the rAd i.m. priming-s.c. boosting strategy, as indicated by the moderate levels of immune responses and protective efficacies. Differences in results may be attributable to various in the employed vaccines. As earlier described, adenoviral vectored vaccines immunized intramuscularly can present antigens via the MHC-I way and elicit Th1-biased responses. However, protein or adenoviral vectored vaccines immunized subcutaneously can present antigens via the MHC-II way and elicit Th2-biased responses. Anti-vector effects are another group of factors that affect protective efficacy that is elicited by a heterologous prime-boost strategy [31, 32, 40]. Through the detection of specific anti-adenovirus antibodies, the highest anti-adenovirus antibody levels were observed in the rAd i.m. priming-s.c. boosting group. These specific antibodies could efficiently neutralize adenoviral vectored vaccines as well as cause antagonistic effects on the final outcomes.

*Schistosoma japonicum*, a genus of complex multicellular pathogen, undergoes six different developmental stages, of which the schistosomulum, adult worm, and egg occur within the definitive host [2]. The specific immune responses against *S. japonicum* infection are complex and have not been clearly elucidated [13]. Previous studies have shown that Th1-biased immune responses play an important role in protecting against infections in a radiation-attenuated cercariae animal model [14, 34]. Furthermore, specific IgG responses may also contribute to an increase in protective efficacy [35]. In the present study, the rAdV i.m. priming-rSjTPI boosting strategy elicited broad spectrum immune responses, which were manifested as higher IgG responses (IgG levels, IgG titers, and IgG avidity), elevated Th1, Th2, and Th17 cytokine levels, as well as produced the highest level of protective efficacy among the three heterologous prime-boost combinations. These results were in agreement with those reported in previous studies.

## Conclusion

In summary, we have developed a suitable immunization strategy, rAdV i.m. priming-rSjTPI boosting strategy, which elicits a high level of protective efficacy against *S. japonicum* infection in mice. However, comparison of different heterologous prime-boost combinations indicated that different factors may be considered when designing a suitable heterologous prime-boost strategy, including types of protective immune responses against infection, characteristics of different vaccines, anti-vector effects, and suitable vaccination routes.

## Additional file

Additional file 1: rSjTPI-specific cytokines (IL-6, IL-10 and IL-17A) induced by a DNA vector (i.m.), Ad vector (i.m.), DNA (i.m.), rAdV (i.m.), DNA (i.m.) + rAdV (i.m.) immunized groups and the control group. (TIF 172 kb)

## Abbreviations

ELISA: Enzyme-linked immunosorbent assay
HRP: Horseradish peroxidase
MHC: Major histocompatibility complex
OD: Odensity
rAdV: Recombinant adenoviral vectored vaccine
rSjTPI: Recombinant triosephosphate isomerase of *Schistosoma japonicum*
