# Supplementary figures and images for: Evaluation of protective efficacy induced by different heterologous prime-boost strategies encoding triosephosphate isomerase against Schistosoma japonicum in mice
Source: Parasit Vectors. 2017 Feb 28;10:111. doi: 10.1186/s13071-017-2036-5 (PMC5330126; doi:10.1186/s13071-017-2036-5)

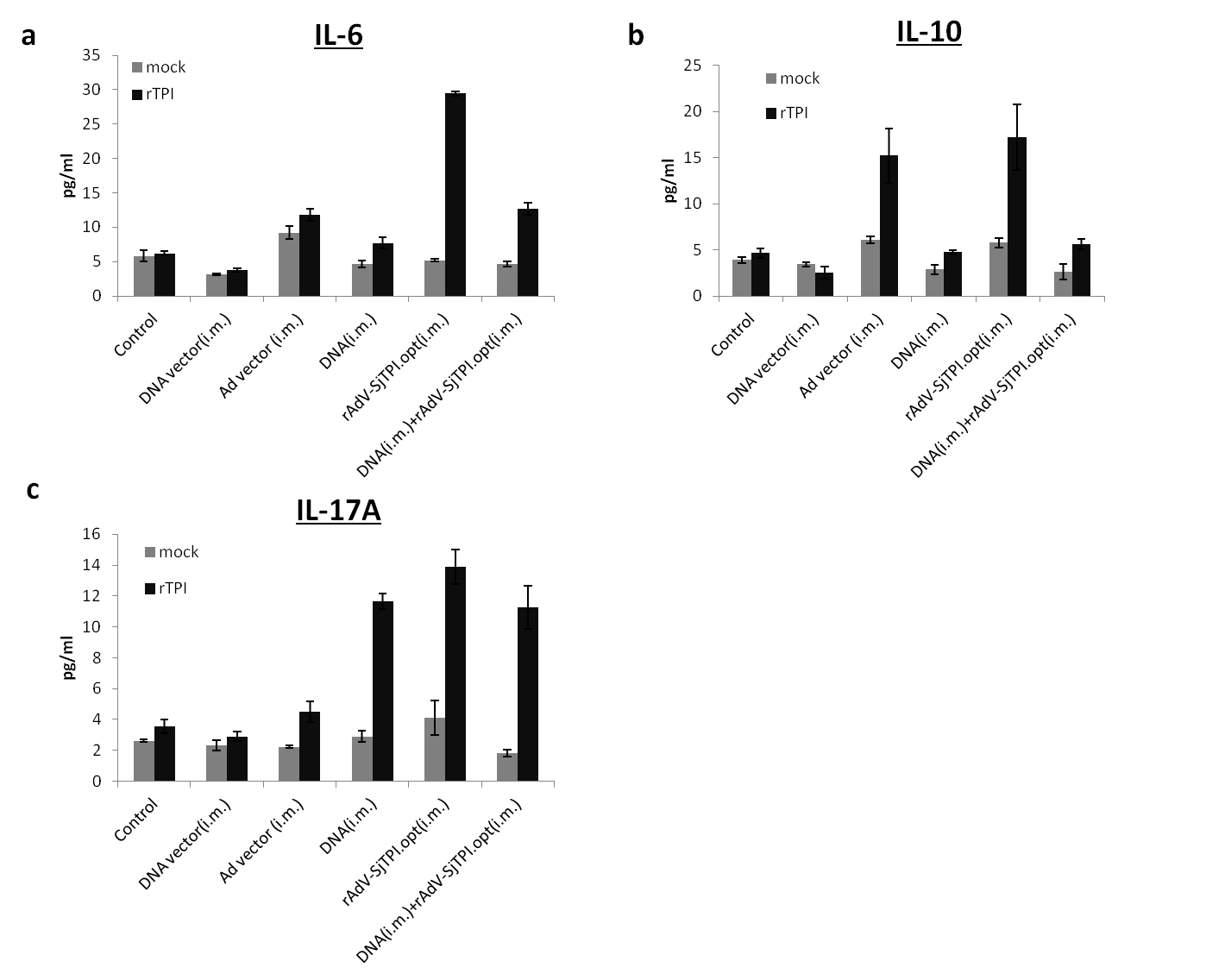

Supplement: Additional file 1: — rSjTPI-specific cytokines (IL-6, IL-10 and IL-17A) induced by a DNA vector (i.m.), Ad vector (i.m.), DNA (i.m.), rAdV (i.m.), DNA (i.m.) + rAdV (i.m.) immunized groups and the control group. (TIF 172 kb) [file 13071_2017_2036_MOESM1_ESM.tif]
